# The association between oxidative balance score and muscular dystrophies

**DOI:** 10.3389/fnut.2024.1465486

**Published:** 2024-09-13

**Authors:** Dupeng Tang, Lanqing Lin, Yixin Zheng

**Affiliations:** ^1^Department of Gastroenterology, The Affiliated People's Hospital of Fujian University of Traditional Chinese Medicine, Fuzhou, China; ^2^Studies of Hospital of Traditional Chinese Medicine of Ningde City, Fujian University of Traditional Chinese Medicine, Fuzhou, China; ^3^Department of Orthopaedics, Shengli Clinical Medical College of Fujian Medical University, Fuzhou, China; ^4^Department of Orthopaedics, Fujian Provincial Hospital, Fuzhou, China

**Keywords:** muscular dystrophies, oxidative balance score, antioxidants, cross-sectional study, NHANES

## Abstract

**Introduction:**

This research utilized data from the NHANES 2011–2018 study to investigate the connection between the Oxidative Balance Score (OBS) and muscular dystrophies.

**Methods:**

This study is a cross-sectional, observational, secondary analysis utilizing data from the NHANES 2011-2018. Spearman’s correlation, chi-square tests, logistic regression, and restricted cubic spline plots were employed for statistical analyses.

**Results:**

This association remained significant after adjustment for various demographic and medical history factors (For continuous OBS: crude model, odds ratio [OR], 0.95, 95% confidence interval [CI:] 0.94, 0.97, *p* < 0.001; Model 1, OR, 0.94, 95% CI: 0.92, 0.96, *p* < 0.001; Model 2, OR, 0.95, 95% CI: 0.93, 0.97, *p* < 0.001; Model 3, OR, 0.95, 95% CI: 0.93, 0.97, *p* < 0.001; In quartile Q4 vs. Q1: Crude model, OR, 0. 42, 95% CI: 0.26, 0.66, *p* < 0.001; Model 1, OR, 0.33, 95% CI: 0.21, 0.52, *p* < 0.001; Model 2, OR, 0.37, 95% CI: 0.23, 0.58, *p* < 0.001; Model 3, OR, 0.38, 95% CI: 0.23, 0.60, *p* < 0.001). Restricted cubic spline (RCS) analysis further supported this inverse relationship, suggesting that OBS values above 10 may confer protection against muscular dystrophies (*p* for overall <0.001, *p* for non-linear = 0.536). However, the relationship between OBS and muscular dystrophies was not statistically significant in the subgroups with education level below high school, presence of cancer, or diabetes (*p* = 0.735, *p* = 0.574, *p* = 0.409, respectively).

**Conclusion:**

The study found a significant inverse correlation between the OBS and muscular dystrophies, suggesting that individuals with higher oxidative balance had a lower risk of developing muscular dystrophies. The study highlights the potential role of oxidative balance in muscular dystrophies prevention and management.

## 1 Introduction

Muscular dystrophies, a condition characterized by age-related loss of skeletal muscle mass, strength, and function, has garnered significant attention as a public health concern worldwide (1, 2). In the United States, the prevalence of muscular dystrophies is estimated to be between 5 and 13% in individuals aged 60 years and older, with a greater burden in racial and ethnic minority groups (3, 4). As the global population ages, the prevalence of muscular dystrophies are expected to rise, posing a substantial burden on healthcare systems, and reducing the quality of life for affected individuals (5, 6). The loss of muscle mass not only impairs physical function but also increases the risk of adverse health outcomes, such as disability, falls, and mortality (7, 8). The consequences of muscular dystrophies extend beyond physical limitations, as it is closely linked to metabolic disorders, such as insulin resistance and type 2 diabetes (9, 10). Furthermore, muscular dystrophy is associated with an increased risk of hospitalization and mortality, highlighting its impact on public health (11).

Several factors contribute to the development of muscular dystrophy, including age-related changes in muscle biology, hormonal alterations, nutritional deficiencies, physical inactivity, and chronic diseases (12–14). Oxidative stress, characterized by an imbalance between the production of reactive oxygen species and the body’s antioxidant defenses, has been proposed as a potential mechanism linking these factors to muscular dystrophy (15, 16). The oxidative balance score (OBS), a measure of the overall oxidative stress status in the body, has been shown to be associated with muscle mass and function in previous studies (17). Xu et al. (18) reported a negative association between OBS and sarcopenia (odd ratio = 0.94, 95%CI 0.92 to 0.96, *p* < 0.001), Cai et al. (19) and Zhao et al. (20) indicated similarly. Therefore, investigating the relationship between oxidative balance score and sarcopenia may provide valuable insights into the pathogenesis of this condition and identify potential targets for intervention.

In this study, we utilize data from the National Health and Nutrition Examination Survey (NHANES) 2011–2018 dataset to examine the association between oxidative balance score and muscular dystrophies. By analyzing this comprehensive dataset, we aim to provide a more accurate understanding of the burden of muscular dystrophies in the United States and its potential links to oxidative stress. The findings of this study may inform future public health interventions and policies aimed at preventing and managing muscular dystrophies, ultimately improving the health and well-being of people with muscular dystrophies.

## 2 Materials and methods

### 2.1 Study design and population

This study is a cross-sectional, observational, secondary analysis utilizing data from the NHANES 2011–2018. NHANES is a continuous, nationwide survey conducted by the National Center for Health Statistics that assesses the health and nutritional status of a representative sample of the non-institutionalized U.S. population. The survey employs a complex, stratified, multistage probability sampling design to ensure that the data collected are generalizable to the U.S. population. The dataset is available online at https://www.cdc.gov/nchs/nhanes/index.htm.

**Figure 1:**
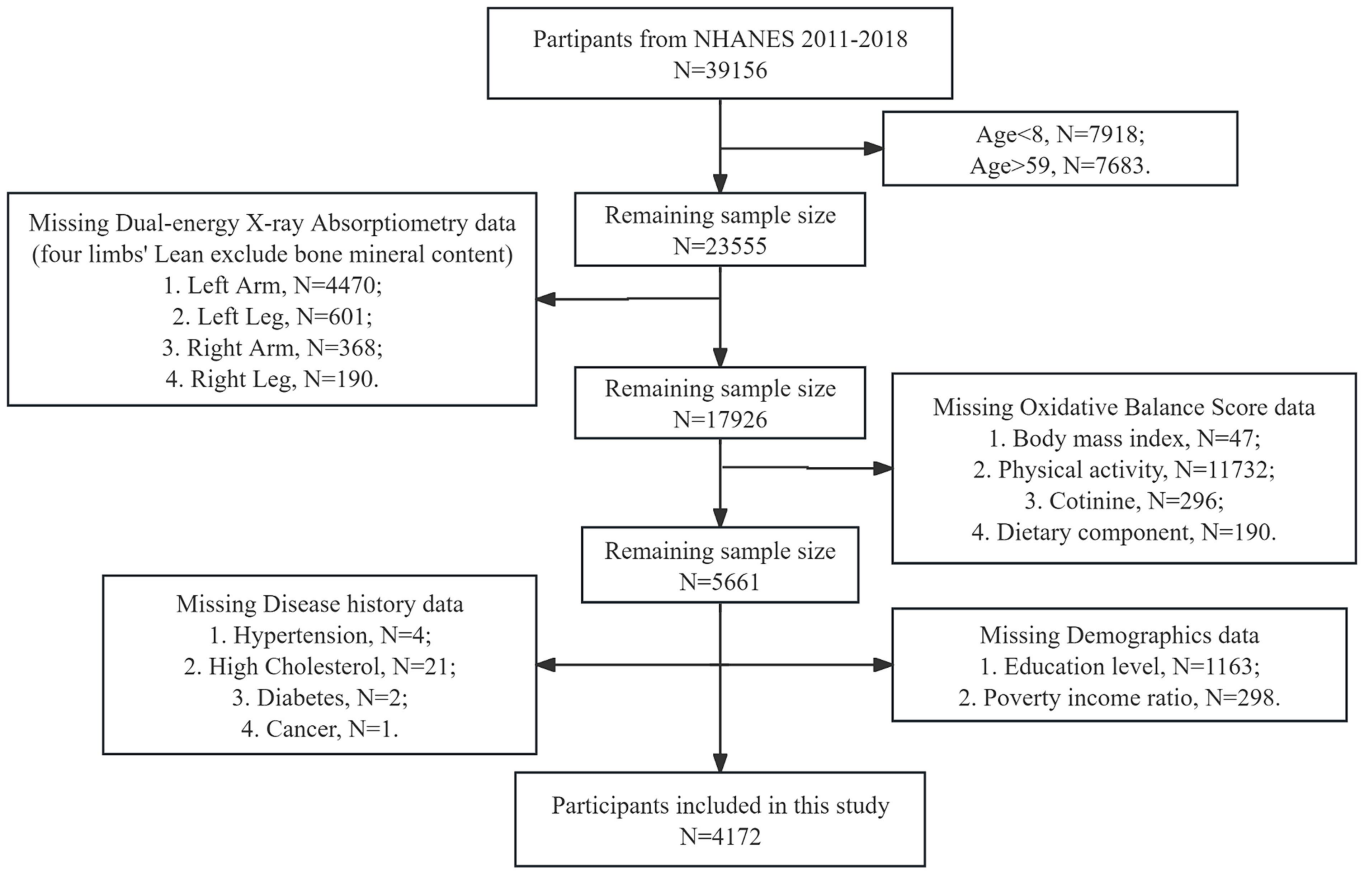
Flow chart of participants selection. NHANES, National Health and Nutrition Examination Survey.

The study population included all individuals aged from 8 to 59 years who participated in the NHANES survey during 2011–2018. The inclusion criterion was based on the availability of complete whole-body dual-energy X-ray absorptiometry (DEXA) data. Participants with missing data on DEXA, body mass index (BMI), OBS, demographic information [race, gender, education level, marital status, and poverty income ratio (PIR)], and medical history (hypertension, high cholesterol, diabetes, and cancer) were excluded from the analysis (Figure 1).

### 2.2 Outcome definitions

The primary outcome of this study was the presence of muscular dystrophies, which was assessed using DEXA whole-body files from the examination data. Prior to analysis, participants were excluded if they were taller than 192.5 cm, weighed more than 136.4 kg, or were pregnant. Appendicular lean mass (ALM) is the sum of the mineral content of the lean body mass of the left and right arms and legs (in grams). Muscular dystrophies were defined based on ALM:BMI <0.789 for men and <0.512 for women, according to criteria established in previous research (21).

### 2.3 Exposure definitions

The exposure indicator in this study was the previously defined OBS (22, 23). The OBS is a composite score that captures the balance of antioxidant and pro-oxidant factors in an individual’s diet and lifestyle. The dietary components were obtained from the Dietary Interview – First Day Total Nutrient Intakes dataset and included dietary fiber, carotene, riboflavin, niacin, total folate, vitamin B12, vitamin C, vitamin E, calcium, magnesium, zinc, copper, selenium, total fat, and iron. Researchers obtained the data by interviewing participants regarding the types and amounts of food they intake during the 24-h period prior to the interview and then calculated the values using the Food and Nutrient Database for Dietary Studies (FNDDS) provided by the U.S. Department of Agriculture. Lifestyle components included physical activity, alcohol, BMI, and cotinine. These components were classified as antioxidants, with the exception of total fat, iron, alcohol, BMI, and cotinine, which were classified as prooxidants. To account for the different scales of the variables, the OBS data were categorized into three tertiles for males and females, respectively (Supplementary Table S1). For the antioxidant components, a score from 0 to 2 was assigned based on the level of intake, with higher values indicating greater antioxidant capacity. Conversely, for the pro-oxidant components, a score ranging from 2 to 0 (alcohol: 1 to 0) was assigned, with higher values indicating a greater pro-oxidant effect. The scores for each component were then summed to obtain the OBS score.

### 2.4 Covariates

The covariates used in this study were categorized into two broad categories: demographic characteristics and medical history. These covariates were selected to account for potential confounding factors that could influence the association between oxidative balance score and muscular dystrophies. Demographic characteristics included age, gender, race, education level, marital status, and PIR. Age was measured as a continuous variable, while gender was categorized as male or female. Race was classified into four categories: Non-Hispanic White, Non-Hispanic Black, Mexican American, and Other Race. Education level (only individuals aged 20 and above) was divided into two groups: below high school and high school or above. Marital status was simplified into married or unmarried. PIR, a measure of economic status, was considered a continuous variable. Medical history was captured through self-reported questionnaire data, assessing the presence of hypertension, high blood cholesterol level, cancer, or diabetes. These conditions were individually categorized as a history of the disease (yes or no) based on the participant’s response to the questionnaire (“have you ever been told by a doctor or other health professional that you had the disease”).

### 2.5 Statistical analysis

Baseline characteristics of the study population were described using muscular dystrophies status (presence or absence) and OBS quartiles. Continuous variables were presented as means and standard deviations, and Spearman’s correlation coefficients were used to assess differences between groups. Categorical variables were described with frequencies and percentages, and chi-square tests were performed to compare group differences. Logistic regression was employed to examine the association between OBS and muscular dystrophies, with adjustment for potential confounding factors. Model 1 was adjusted for age, gender, and race. Model 2 included additional adjustments for education level, marital status, and PIR. Model 3 further adjusted for hypertension, high cholesterol, cancer, and diabetes. Restricted cubic spline (RCS) plots were used to examine the non-linear relationship between muscular dystrophies and OBS. Subgroup analyses were conducted to evaluate interactions between muscular dystrophies and OBS in different populations. All data were weighted using NHANES standard weights to ensure representativeness of the results. Statistical analyses were performed using R software version 4.3.2. Statistical significance was defined as a two-tailed *p*-value <0.05.

## 3 Results

### 3.1 Baseline characteristics

A total of 4,172 participants were included in the study, of whom 320 (7.7%) met the criteria for muscular dystrophies and 3,852 (92.3%) were muscular non-dystrophies (Table 1). The mean age of the participants was 38.4 years, with those diagnosed with muscular dystrophies being older (42.7 years) than those without muscular dystrophies (38.0 years, *p* < 0.001). There were more males (56.0%) and a higher proportion of individuals with a high school education or higher (83.3%) in the muscular non-dystrophies group (*p* = 0.977, *p* < 0.001, respectively). In addition, the Mexican American (15.5%) and non-Hispanic Black (19.9%) races were less prevalent in the muscular non-dystrophies group (*p* < 0.001). Furthermore, participants with muscular dystrophies had a lower PIR (2.04) compared to those without (2.40, *p* < 0.001). The prevalence of cancer, hypertension, high cholesterol, and diabetes was higher in the muscular non-dystrophies group (*p* = 0.006, *p* < 0.001, *p* < 0.001, *p* < 0.001, respectively). OBS (mean: total OBS, 20.0; dietary OBS, 16.3; lifestyle OBS, 3.66) was significantly lower in the muscular dystrophies group compared to the muscular non-dystrophies group (total OBS, 20.1 vs. 18.4, *p* < 0.001; dietary OBS, 16.4 vs. 15.1, *p* = 0.002; lifestyle OBS, 3.70 vs. 3.26, *p* < 0.001). Table 2 shows the baseline characteristics of all participants grouped by quartile of OBS. Participants in the Q4 group had a decreasing trend in the prevalence of muscular dystrophies (5.6%) compared to those in the Q3 group (7.23%), Q2 group (8.58%), Q1 group (9.34%). The age distribution was slightly higher in Q3 and Q4 groups compared to Q1 and Q2 groups. Regarding gender, there was a higher percentage of males in the Q4 group compared to the Q1 group. Educational attainment was higher in the Q4 group, with fewer individuals having less than a high school education. The PIR was highest in the Q4 group, indicating a higher economic status. The prevalence of cancer, hypertension, high cholesterol, and diabetes was lower in the Q4 group than in the Q1 group. Baseline characteristics based on the quartile dietary and lifestyle OBS are summarized in Supplementary Tables S2, S3, respectively.

**Table 1:** Baseline characteristics of all participants by sarcopenia.

Variables	Overall *N* = 4,172	Muscular dystrophy N = 320	Muscular non-dystrophy N = 3,852	*p*-value
Age [mean (SD)]	38.4 (11.5)	42.7 (11.9)	38.0 (11.4)	<0.001
Gender				0.977
Female	1835 (44.0%)	140 (43.8%)	1,695 (44.0%)	
Male	2,337 (56.0%)	180 (56.2%)	2,157 (56.0%)	
Education				<0.001
Below high school	696 (16.7%)	84 (26.2%)	612 (15.9%)	
High School or above	3,476 (83.3%)	236 (73.8%)	3,240 (84.1%)	
Race				<0.001
Mexican American	645 (15.5%)	125 (39.1%)	520 (13.5%)	
Non-Hispanic Black	829 (19.9%)	16 (5.00%)	813 (21.1%)	
Non-Hispanic White	1775 (42.5%)	104 (32.5%)	1,671 (43.4%)	
Others	923 (22.1%)	75 (23.4%)	848 (22.0%)	
Marital status				0.438
No	1,656 (39.7%)	120 (37.5%)	1,536 (39.9%)	
Yes	2,516 (60.3%)	200 (62.5%)	2,316 (60.1%)	
Poverty Income Ratio [mean (SD)]	2.38 (1.58)	2.04 (1.42)	2.40 (1.59)	<0.001
Cancer				0.006
No	4,010 (96.1%)	298 (93.1%)	3,712 (96.4%)	
Yes	162 (3.88%)	22 (6.88%)	140 (3.63%)	
Hypertension				<0.001
No	3,168 (75.9%)	211 (65.9%)	2,957 (76.8%)	
Yes	1,004 (24.1%)	109 (34.1%)	895 (23.2%)	
High cholesterol				<0.001
No	3,188 (76.4%)	209 (65.3%)	2,979 (77.3%)	
Yes	984 (23.6%)	111 (34.7%)	873 (22.7%)	
Diabetes				<0.001
No	3,813 (91.4%)	258 (80.6%)	3,555 (92.3%)	
Yes	359 (8.60%)	62 (19.4%)	297 (7.71%)	
OBS [mean (SD)]	20.0 (7.44)	20.1 (7.44)	18.4 (7.25)	<0.001
Dietary OBS (mean [SD])	16.3 (7.15)	16.4 (7.16)	15.1 (6.94)	0.002
Lifestyle OBS (mean [SD])	3.66 (1.40)	3.70 (1.40)	3.26 (1.37)	<0.001

SD, standard deviation. OBS, oxidative balance score.

**Table 2:** Baseline characteristics of all participants by oxidative balance score.

Variables	Oxidative balance score				*p*-value
	Q1 *N* = 985	Q2 *N* = 1,014	Q3 *N* = 1,190	Q4 *N* = 983	
Muscular dystrophies					0.01
No	893 (90.7%)	927 (91.4%)	1,104 (92.8%)	928 (94.4%)	
Yes	92 (9.34%)	87 (8.58%)	86 (7.23%)	55 (5.60%)	
Age [mean (SD)]	37.6 (11.5)	39.0 (11.6)	38.6 (11.4)	38.1 (11.5)	0.03
Gender					0.055
Female	443 (45.0%)	466 (46.0%)	530 (44.5%)	396 (40.3%)	
Male	542 (55.0%)	548 (54.0%)	660 (55.5%)	587 (59.7%)	
Education					0.001
Below high school	202 (20.5%)	174 (17.2%)	174 (14.6%)	146 (14.9%)	
High School or above	783 (79.5%)	840 (82.8%)	1,016 (85.4%)	837 (85.1%)	
Race					<0.001
Mexican American	106 (10.8%)	143 (14.1%)	202 (17.0%)	194 (19.7%)	
Non-Hispanic Black	259 (26.3%)	224 (22.1%)	198 (16.6%)	148 (15.1%)	
Non-Hispanic White	415 (42.1%)	423 (41.7%)	522 (43.9%)	415 (42.2%)	
Others	205 (20.8%)	224 (22.1%)	268 (22.5%)	226 (23.0%)	
Marital status					<0.001
No	472 (47.9%)	395 (39.0%)	439 (36.9%)	350 (35.6%)	
Yes	513 (52.1%)	619 (61.0%)	751 (63.1%)	633 (64.4%)	
Poverty income ratio [mean (SD)]	2.02 (1.49)	2.46 (1.59)	2.51 (1.59)	2.49 (1.59)	<0.001
Cancer					0.135
No	936 (95.0%)	976 (96.3%)	1,154 (97.0%)	944 (96.0%)	
Yes	49 (4.97%)	38 (3.75%)	36 (3.03%)	39 (3.97%)	
Hypertension					0.01
No	727 (73.8%)	750 (74.0%)	910 (76.5%)	781 (79.5%)	
Yes	258 (26.2%)	264 (26.0%)	280 (23.5%)	202 (20.5%)	
High cholesterol					0.01
No	786 (79.8%)	752 (74.2%)	890 (74.8%)	760 (77.3%)	
Yes	199 (20.2%)	262 (25.8%)	300 (25.2%)	223 (22.7%)	
Diabetes					0.064
No	899 (91.3%)	908 (89.5%)	1,094 (91.9%)	912 (92.8%)	
Yes	86 (8.73%)	106 (10.5%)	96 (8.07%)	71 (7.22%)	

SD, standard deviation.

### 3.2 Relationship between OBS and muscular dystrophies

The results of multivariate logistic regression suggested a significant correlation between the dependent variable muscular dystrophies and the independent variable OBS (Table 3). The dietary and lifestyle OBS likewise demonstrated a significant association with muscular dystrophies (Supplementary Tables S4, S5, respectively). In the crude model, a significant inverse association was observed with an odds ratio (OR) of 0.95 (95% confidence interval [CI]: 0.94, 0.97), indicating that higher OBS was associated with a lower risk of muscular dystrophies (*p* < 0.001). After adjusting for age, gender, and race in model 1, the association remained significant with an OR of 0.94 (95% CI: 0.92, 0.96) (*p* < 0.001). Further adjustment for education level, marital status, and PIR in model 2 resulted in an OR of 0.95 (95% CI: 0.93, 0.97) (*p* < 0.001). Model 3, which included additional adjustments for hypertension, high cholesterol, cancer, and diabetes, also showed a significant inverse association with an OR of 0.95 (95% CI: 0.93, 0.97) (*p* < 0.001). In quartile Q4, the OR for muscular dystrophies was 0.42 (95% CI: 0.26, 0.66) in the crude model, 0.33 (95% CI: 0.21, 0.52) in model 1, 0.37 (95% CI: 0.23, 0.58) in model 2, and 0.38 (95% CI: 0.24, 0.60) in model 3, with *p*-values for trend <0.001 in all models.

**Table 3:** Association of oxidative balance score with muscular dystrophies.

Muscular dystrophies	Odds ratio (95%CI); *p*-value							
	Crude model		Model 1		Model 2		Model 3	
Continuous	0.95 (0.94, 0.97)	<0.001	0.94 (0.92, 0.96)	<0.001	0.95 (0.93, 0.97)	<0.001	0.95 (0.93, 0.97)	<0.001
Q1	1.00 (ref)		1.00 (ref)		1.00 (ref)		1.00 (ref)	
Q2	0.84 (0.59, 1.20)	0.341	0.75 (0.51, 1.08)	0.125	0.83 (0.58, 1.20)	0.316	0.84 (0.58, 1.22)	0.359
Q3	0.67 (0.47, 0.96)	0.029	0.55 (0.38, 0.80)	0.002	0.62 (0.43, 0.91)	0.014	0.64 (0.44, 0.94)	0.025
Q4	0.42 (0.26, 0.66)	<0.001	0.33 (0.21, 0.52)	<0.001	0.37 (0.23, 0.58)	<0.001	0.38 (0.24, 0.60)	<0.001
*p* for trend		<0.001		<0.001		<0.001		<0.001

The OBS was converted from a continuous variable to a categorical variable (quartiles). Data are presented as OR (95% CI). Crude model was adjusted with no covariates. Model 1 was adjusted for age, gender, and race. Model 2 included additional adjustments for education level, marital status, and PIR. Model 3 further adjusted for hypertension, high cholesterol, cancer, and diabetes.

### 3.3 RCS analysis

**Figure 2:**
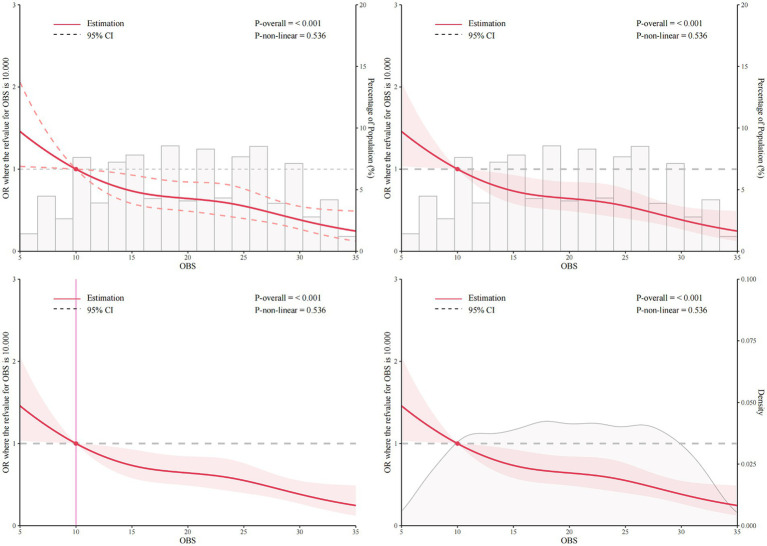
Restricted cubic spline (RCS) analysis of the association between OBS and muscular dystrophies with number, bar chart of proportion, and density. The association was adjusted for age, gender, race, education level, marital status, poverty income ratio, hypertension, high cholesterol, cancer, and diabetes. The median OBS was chosen as the reference. RCS, restricted cubic spline; OBS, oxidative balance score.

The RCS analysis was conducted to assess the relationship between OBS and muscular dystrophies, adjusting for covariates in model 3. The analysis was stratified by the overall population as well as male and female groups to explore the potential non-linear associations. The RCS curve shown in Figure 2 indicated a significant association between OBS and muscular dystrophies in the overall population (*p*-overall < 0.001). However, there was no evidence of a non-linear association (*p* for non-linear = 0.536). This suggests that with adjustment for covariates in model 3, as OBS increases, the odds of muscular dystrophies decrease linearly. The inflection point at OBS = 10.0 indicates that an OBS greater than 10 may have a protective effect on the occurrence of muscular dystrophies. In the male population, RCS analysis indicated a linear relationship between OBS and muscular dystrophies (*p*-overall = 0.002, *p* for non-linear = 0.194, Supplementary Figure S1). Similar results were observed in the female population (*p*-overall < 0.001, *p* for non-linear = 0.500, Supplementary Figure S2).

### 3.4 Subgroup analysis

**Figure 3:**
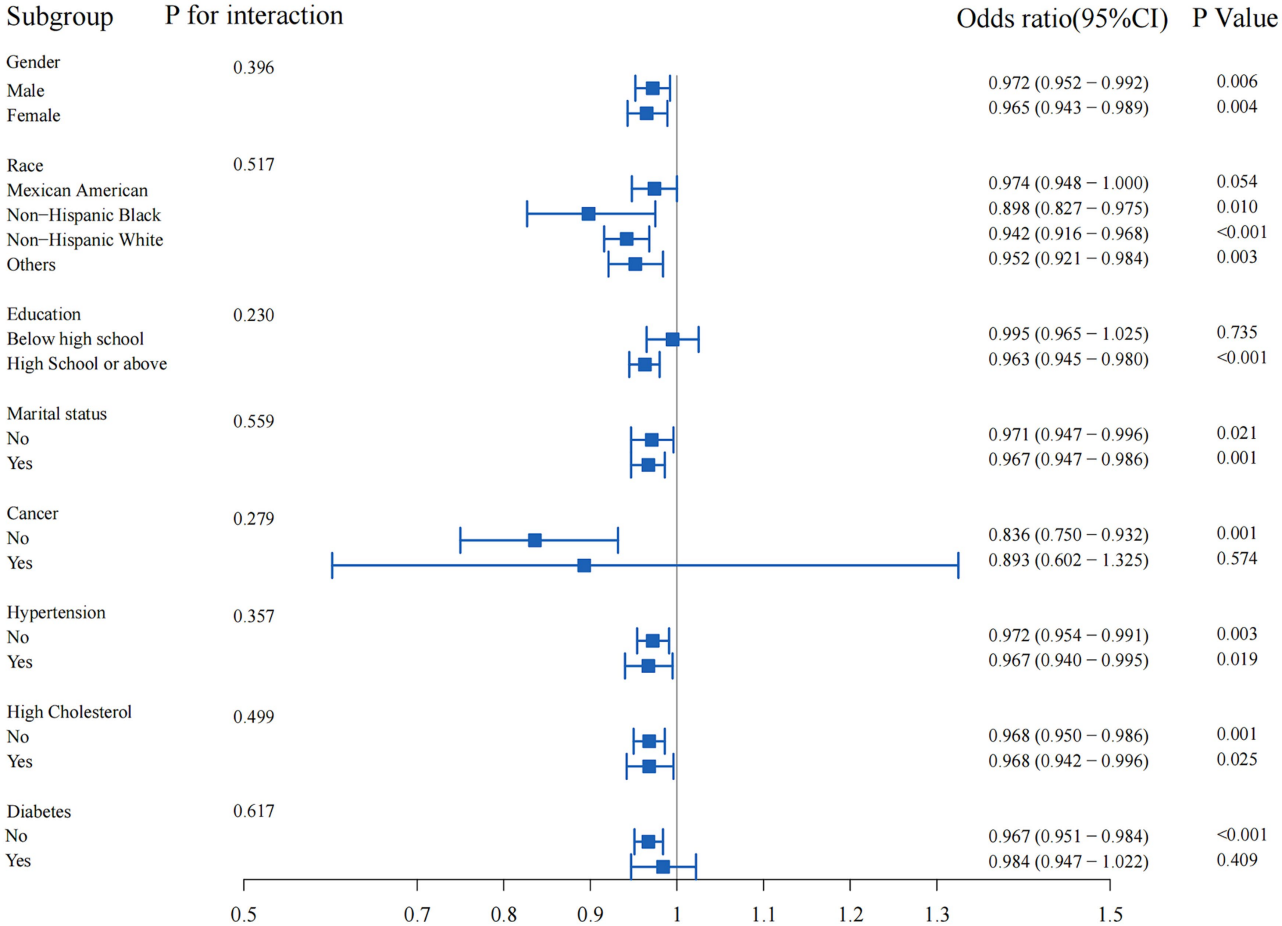
Subgroup analyses of the association between oxidative balance score and muscular dystrophies.

Subgroup analysis was performed to evaluate the potential interaction between OBS and muscular dystrophies in several subgroups, including gender, race, education level, marital status, hypertension, high cholesterol, cancer, and diabetes (Figure 3). Overall, there was no statistically significant interaction between OBS and muscular dystrophies in any of the subgroups (all *p*-values for interaction >0.05). However, the association between OBS and muscular dystrophies was not statistically significant in the subgroups with education level below high school, presence of cancer, or diabetes (*p* = 0.735, *p* = 0.574, *p* = 0.409, respectively).

## 4 Discussion

This study sought to explore the association between the OBS and muscular dystrophies among a sample of participants from the NHANES 2011–2018. The findings revealed a significant inverse relationship between OBS and muscular dystrophies in the US population, indicating that higher oxidative balance was associated with a lower risk of muscular dystrophies. This relationship remained significant after adjusting for several covariates, suggesting that oxidative balance may play a crucial role in the development and progression of muscular dystrophies (19).

The observed inverse relationship between OBS and muscular dystrophies is consistent with previous findings linking oxidative stress to age-related muscle loss (18–20, 24). Oxidative stress, characterized by an imbalance between the production of reactive oxygen species and the body’s antioxidant defenses, has been proposed as a potential mechanism linking various factors, such as age-related changes in muscle biology, hormonal changes, nutritional deficiencies, physical inactivity, and chronic diseases, to muscular dystrophies (12, 15, 25, 26). OBS, a measure of indirect oxidative stress status in the body, has been shown in previous studies to be associated with muscle mass and function (27, 28). Therefore, the inverse association observed in this study underscores the potential role of oxidative balance in the pathogenesis of muscular dystrophies and highlights the importance of interventions targeting oxidative balance to prevent and treat muscular dystrophies. In addition, the association of dietary OBS and lifestyle OBS with muscular dystrophies are also noteworthy. Dietary OBS, which captures the balance of antioxidant and pro-oxidant factors in an individual’s diet, has been shown in previous studies to be associated with muscle mass and function (29, 30). Similarly, Lifestyle OBS, which reflects the balance of antioxidant and pro-oxidant factors in an individual’s lifestyle, has also been associated with muscle health (31). In this study, both Dietary OBS and Lifestyle OBS showed significant associations with muscular dystrophies, further supporting the role of oxidative balance in the development of muscular dystrophies.

Furthermore, evidence from previous research supports the notion that antioxidant factors can play a protective role against muscular dystrophies. For instance, dietary antioxidants such as vitamin C, vitamin E, and carotenoids have been shown to have a beneficial effect on muscle mass and strength (25, 32). These antioxidants can reduce oxidative stress and protect muscle fibers from damage, thereby potentially preventing the development of sarcopenia (32–34). Additionally, physical activity, which is classified as an antioxidant factor in the OBS, has been linked to increased muscle mass and strength, probably through its anti-inflammatory and antioxidant effects (35, 36). On the other hand, pro-oxidant factors, such as certain types of dietary fat, alcohol, and tobacco, have been associated with an increased risk of sarcopenia (37, 38). These factors can exacerbate oxidative stress and lead to muscle damage and loss (39). Oxidative stress could lead to an imbalance in protein synthesis and degradation, thereby increasing muscle catabolism and protein breakdown (18). Furthermore, oxidative stress may disrupt calcium homeostasis within muscle cells, impairing muscle contraction and relaxation functions, and ultimately contributing to the development of muscular dystrophies (29). Therefore, maintaining a balanced diet and lifestyle, rich in antioxidants and low in pro-oxidants, may be an effective strategy for preventing muscular dystrophies.

The RCS analysis further supported the inverse relationship between OBS and muscular dystrophies, indicating a linear decrease in the odds of muscular dystrophies as OBS increased. This finding suggests that higher OBS values may have a protective exogenous effect against the occurrence of muscular dystrophies. The inflection point of the RCS plots further underscores the potential protective effect of an OBS greater than 10 in reducing the risk of muscular dystrophies. This finding is particularly meaningful as it provides a potential target for interventions aimed at improving oxidative balance to reduce the risk of muscular dystrophies.

Subgroup analysis revealed no statistically significant interaction between OBS and muscular dystrophies in various demographic and medical history subgroups. However, the association between OBS and muscular dystrophies were not statistically significant in the subgroups with education level below high school, presence of cancer, or diabetes. This could be due to the potential influence of the categorical nature of these variables on the association between OBS and muscular dystrophies. First, the potential influence of socioeconomic status on dietary and lifestyle choices may have obscured the relationship between OBS and muscular dystrophies (18). Lower socioeconomic status is associated with less access to nutritious foods and health-promoting activities, which may affect oxidative balance and muscle health (40). In addition, individuals with lower levels of education may have less awareness or access to health information and resources, further complicating the association between OBS and muscular dystrophies (18). In the subgroup with a history of cancer, the potential effects of cancer and its treatment on muscle health and oxidative balance may have confounded the observed association between OBS and muscular dystrophies (8). Cancer and cancer therapy can lead to significant changes in muscle mass and function, as well as changes in antioxidant status, which may explain the lack of a significant association in this subgroup (41, 42). In the subgroup with diabetes, the complex interplay of metabolic factors associated with diabetes, such as insulin resistance and inflammation, may have influenced the relationship between OBS and muscular dystrophies (9, 43). Furthermore, it has been reported that ischemic heart disease and frailty may be associated with the body’s antioxidant function (44, 45). These metabolic changes may independently affect muscle health and oxidative balance, potentially masking the impact of OBS on muscular dystrophies (9). This nuanced finding highlights the complexity of the relationship between oxidative balance and muscular dystrophies and underscores the need for further investigation of potential interactions in specific subgroups and different levels of molecular organization.

Limitations of this study include reliance on self-reported data, potential unmeasured confounders (such as nutritional supplements), and the cross-sectional nature of the analysis, which limits causal inference. In future research, longitudinal studies and intervention trials may provide further insight into the causal relationship between oxidative balance and muscular dystrophies. In addition, exploring the mechanistic pathways underlying the observed associations and investigating the impact of lifestyle interventions on oxidative balance and muscular dystrophies could improve our understanding of these complex interactions and inform targeted interventions to optimize muscle health in at-risk populations.

## 5 Conclusion

In conclusion, this study provides evidence of a significant inverse association between OBS and muscular dystrophies in the US population, being significant in the group with a better oxidative balance. The findings highlight the importance of interventions targeting oxidative balance, such as dietary and lifestyle modifications, to promote muscle health and reduce the risk of muscular dystrophies. Future longitudinal studies and intervention trials are needed to further explore the causal relationship between oxidative balance and muscular dystrophies and to inform the development of effective public health strategies for the prevention and treatment of muscular dystrophies.

## Data Availability

The datasets presented in this study can be found in online repositories. The names of the repository/repositories and accession number(s) can be found at: https://www.cdc.gov/nchs/nhanes/index.htm.
