# Supplementary material for: The association between oxidative balance score and muscular dystrophies
Source: Front Nutr. 2024 Sep 13;11:1465486. doi: 10.3389/fnut.2024.1465486 (PMC11427402; doi:10.3389/fnut.2024.1465486)
Supplement: Supplementary file 1 [file Data_Sheet_1.docx]

Supplementary Material

**The Association Between Oxidative Balance Score and Muscular Dystrophies**

# Supplementary Data

Supplementary Material include supplementary table 1 – supplementary table 5 and supplementary figure 1 – supplementary figure 2. The above documents were submitted separately.

# Supplementary Tables

**Supplemental table 1.** Oxidative Balance Score assignment scheme.

| **OBS components** | Property | Scoring assignment | | | | | |
| --- | --- | --- | --- | --- | --- | --- | --- |
|  |  | Male | | | Female | | |
|  |  | 0 | 1 | 2 | 0 | 1 | 2 |
| **Dietary Components** |  |  |  |  |  |  |  |
| Dietary fiber (g/d) | Anti-oxidant | <12.6 | 12.6-21.2 | >21.2 | <10.10 | 10.10-17.2 | >17.2 |
| Carotene (RE/d) | Anti-oxidant | <109.25 | 109.25-356.5 | >356.5 | <105.5 | 105.5-418 | >418 |
| Riboflavin (mg/d) | Anti-oxidant | <1.72 | 1.72-2.73 | >2.73 | <1.30 | 1.30-2.04 | >2.04 |
| Niacin (mg/d) | Anti-oxidant | <23.83 | 23.83-36.56 | >36.56 | <16.23 | 16.23-24.53 | >24.53 |
| Vitamin B6 (mg/d) | Anti-oxidant | <1.69 | 1.69-2.78 | >2.78 | <1.19 | 1.19-1.92 | >1.92 |
| Total folate (mcg/d) | Anti-oxidant | <312 | 312-512 | >512 | <238 | 238-377 | >377 |
| Vitamin B12 (mcg/d) | Anti-oxidant | <3.40 | 3.40-7.08 | >7.08 | <2.21 | 2.21-4.57 | >4.57 |
| Vitamin C (mg/d) | Anti-oxidant | <25.3 | 25.3-85.4 | >85.4 | <25.2 | 25.2-74.4 | >74.4 |
| Vitamin E (ATE)(mg/d) | Anti-oxidant | <6.23 | 6.23-10.69 | >10.69 | <5.2 | 5.2-9.03 | >9.03 |
| Calcium (mg/d) | Anti-oxidant | <741 | 741-1252 | >1252 | <595 | 595-976 | >976 |
| Magnesium (mg/d) | Anti-oxidant | <257 | 257-387 | >387 | <203 | 203-296 | >296 |
| Zinc (mg/d) | Anti-oxidant | <9.50 | 9.50-15.15 | >15.15 | <6.66 | 6.66-10.62 | >10.62 |
| Copper (mg/d) | Anti-oxidant | <1.66 | 1.50-1.66 | >1.50 | <0.79 | 0.79-1.20 | >1.20 |
| Selenium (mcg/d) | Anti-oxidant | <105.4 | 105.4-160.8 | >160.80 | <74.1 | 74.1-113.00 | >113.00 |
| Total fat (g/d) | Pro-oxidant | >114.2 | 73.52-114.2 | <73.52 | >86.02 | 56.02-86.02 | <56.02 |
| Iron (mg/d) | Pro-oxidant | >18.97 | 11.89-18.97 | <11.89 | >13.63 | 8.80-13.63 | <8.80 |
| **Lifestyle Components** |  |  |  |  |  |  |  |
| Physical activity (MET-minute/week) | Anti-oxidant | <240 | 240-1260 | >1260 | <297.5 | 297.5-840 | >840 |
| Alcohol (g/d) | Pro-oxidant | >30 | 0-30 | None | >15 | 0-15 | None |
| Body mass index (kg/m2) | Pro-oxidant | >30.40 | 25.8-30.4 | <25.8 | >32.2 | 25.4-32.2 | <25.4 |
| Cotinine (ng/mL) | Pro-oxidant | >1.490 | 0.031-1.490 | <0.031 | >0.242 | 0.016-0.242 | <0.016 |
| Abbreviations: oxidative balance score, OBS; RE, retinol equivalent; ATE, alpha-tocopherol equivalent; MET, metabolic equivalent. | | | | | | | |

**Supplemental table 2.** Baseline characteristics of all participants by Dietary Oxidative Balance Score.

| Variables | Dietary Oxidative Balance Score | | | | *p*-Value |
| --- | --- | --- | --- | --- | --- |
|  | Q1 N=1079 | Q2 N=1035 | Q3 N=1163 | Q4 N=995 |  |
| Muscular Dystrophies |  |  |  |  | 0.01 |
| No | 893 (90.7%) | 927 (91.4%) | 1104 (92.8%) | 928 (94.4%) |  |
| Yes | 92 (9.34%) | 87 (8.58%) | 86 (7.23%) | 55 (5.60%) |  |
| Age | 37.5 (11.6) | 38.9 (11.5) | 38.7 (11.5) | 38.3 (11.4) | 0.033 |
| Gender |  |  |  |  | 0.02 |
| Female | 495 (45.9%) | 472 (45.6%) | 472 (44.4%) | 396 (39.8%) |  |
| Male | 584 (54.1%) | 563 (54.4%) | 591 (55.6%) | 599 (60.2%) |  |
| Education |  |  |  |  | 0.002 |
| Below high school | 218 (20.2%) | 174 (16.8%) | 158 (14.9%) | 146 (14.7%) |  |
| High School or above | 861 (79.8%) | 861 (83.2%) | 905 (85.1%) | 849 (85.3%) |  |
| Race |  |  |  |  | <0.001 |
| Mexican American | 124 (11.5%) | 150 (14.5%) | 185 (17.4%) | 186 (18.7%) |  |
| Non-Hispanic Black | 268 (24.8%) | 229 (22.1%) | 169 (15.9%) | 163 (16.4%) |  |
| Non-Hispanic White | 449 (41.6%) | 430 (41.5%) | 473 (44.5%) | 423 (42.5%) |  |
| Others | 238 (22.1%) | 226 (21.8%) | 236 (22.2%) | 223 (22.4%) |  |
| Marital status |  |  |  |  | <0.001 |
| No | 506 (46.9%) | 397 (38.4%) | 401 (37.7%) | 352 (35.4%) |  |
| Yes | 573 (53.1%) | 638 (61.6%) | 662 (62.3%) | 643 (64.6%) |  |
| Poverty Income Ratio | 2.07 (1.51) | 2.45 (1.58) | 2.53 (1.60) | 2.47 (1.59) | <0.001 |
| Cancer |  |  |  |  | 0.192 |
| No | 1031 (95.6%) | 990 (95.7%) | 1033 (97.2%) | 956 (96.1%) |  |
| Yes | 48 (4.45%) | 45 (4.35%) | 30 (2.82%) | 39 (3.92%) |  |
| Hypertension |  |  |  |  | 0.272 |
| No | 809 (75.0%) | 771 (74.5%) | 827 (77.8%) | 761 (76.5%) |  |
| Yes | 270 (25.0%) | 264 (25.5%) | 236 (22.2%) | 234 (23.5%) |  |
| High Cholesterol |  |  |  |  | 0.012 |
| No | 862 (79.9%) | 766 (74.0%) | 805 (75.7%) | 755 (75.9%) |  |
| Yes | 217 (20.1%) | 269 (26.0%) | 258 (24.3%) | 240 (24.1%) |  |
| Diabetes |  |  |  |  | 0.14 |
| No | 990 (91.8%) | 928 (89.7%) | 981 (92.3%) | 914 (91.9%) |  |
| Yes | 89 (8.25%) | 107 (10.3%) | 82 (7.71%) | 81 (8.14%) |  |

**Supplemental table 3.** Baseline characteristics of all participants by Lifestyle Oxidative Balance Score.

| Variables | Lifestyle Oxidative Balance Score | | | | *p*-Value |
| --- | --- | --- | --- | --- | --- |
|  | Q1 N=876 | Q2 N=1012 | Q3 N=1105 | Q4 N=1179 |  |
| Muscular Dystrophies |  |  |  |  | <0.001 |
| No | 781 (89.2%) | 921 (91.0%) | 1031 (93.3%) | 1119 (94.9%) |  |
| Yes | 95 (10.8%) | 91 (8.99%) | 74 (6.70%) | 60 (5.09%) |  |
| Age | 39.9 (11.4) | 39.0 (11.3) | 38.0 (11.4) | 36.9 (11.7) | <0.001 |
| Gender |  |  |  |  | 0.073 |
| Female | 352 (40.2%) | 448 (44.3%) | 496 (44.9%) | 539 (45.7%) |  |
| Male | 524 (59.8%) | 564 (55.7%) | 609 (55.1%) | 640 (54.3%) |  |
| Education |  |  |  |  | 0.124 |
| Below high school | 152 (17.4%) | 147 (14.5%) | 182 (16.5%) | 215 (18.2%) |  |
| High School or above | 724 (82.6%) | 865 (85.5%) | 923 (83.5%) | 964 (81.8%) |  |
| Race |  |  |  |  | <0.001 |
| Mexican American | 97 (11.1%) | 133 (13.1%) | 181 (16.4%) | 234 (19.8%) |  |
| Non-Hispanic Black | 242 (27.6%) | 200 (19.8%) | 215 (19.5%) | 172 (14.6%) |  |
| Non-Hispanic White | 390 (44.5%) | 481 (47.5%) | 452 (40.9%) | 452 (38.3%) |  |
| Others | 147 (16.8%) | 198 (19.6%) | 257 (23.3%) | 321 (27.2%) |  |
| Marital status |  |  |  |  | 0.001 |
| No | 397 (45.3%) | 393 (38.8%) | 429 (38.8%) | 437 (37.1%) |  |
| Yes | 479 (54.7%) | 619 (61.2%) | 676 (61.2%) | 742 (62.9%) |  |
| Poverty Income Ratio | 2.21 (1.59) | 2.37 (1.56) | 2.46 (1.56) | 2.44 (1.60) | 0.002 |
| Cancer |  |  |  |  | 0.122 |
| No | 842 (96.1%) | 967 (95.6%) | 1055 (95.5%) | 1146 (97.2%) |  |
| Yes | 34 (3.88%) | 45 (4.45%) | 50 (4.52%) | 33 (2.80%) |  |
| Hypertension |  |  |  |  | <0.001 |
| No | 546 (62.3%) | 741 (73.2%) | 876 (79.3%) | 1005 (85.2%) |  |
| Yes | 330 (37.7%) | 271 (26.8%) | 229 (20.7%) | 174 (14.8%) |  |
| High Cholesterol |  |  |  |  | <0.001 |
| No | 632 (72.1%) | 739 (73.0%) | 871 (78.8%) | 946 (80.2%) |  |
| Yes | 244 (27.9%) | 273 (27.0%) | 234 (21.2%) | 233 (19.8%) |  |
| Diabetes |  |  |  |  | <0.001 |
| No | 775 (88.5%) | 914 (90.3%) | 1010 (91.4%) | 1114 (94.5%) |  |
| Yes | 101 (11.5%) | 98 (9.68%) | 95 (8.60%) | 65 (5.51%) |  |

**Supplemental table 4.** Association of Dietary Oxidative Balance Score with Muscular Dystrophies.

| Muscular Dystrophies | Odds Ratio (95%CI); *p*-value | | | | | | | |  |
| --- | --- | --- | --- | --- | --- | --- | --- | --- | --- |
|  | Crude model | | Model 1 | | Model 2 | | Model 3 | |  |
| Continuous | 0.96 (0.94, 0.98) | <0.001 | 0.95 (0.93, 0.97) | <0.001 | 0.96 (0.94, 0.98) | <0.001 | 0.96 (0.94, 0.98) | <0.001 | |
| Q1 | 1.00 (ref) | | 1.00 (ref) | | 1.00 (ref) | | 1.00 (ref) | |  |
| Q2 | 0.82 (0.57, 1.18) | 0.281 | 0.74 (0.51, 1.08) | 0.114 | 0.81 (0.56, 1.16) | 0.242 | 0.80 (0.56, 1.14) | 0.216 | |
| Q3 | 0.73 (0.52, 1.01) | 0.059 | 0.61 (0.42, 0.87) | 0.007 | 0.67 (0.46, 0.97) | 0.033 | 0.69 (0.48, 1.01) | 0.055 | |
| Q4 | 0.53 (0.34, 0.84) | 0.008 | 0.44 (0.28, 0.70) | <0.001 | 0.49 (0.31, 0.77) | 0.003 | 0.49 (0.31, 0.77) | 0.003 | |
| *p* for trend |  | 0.004 |  | <0.001 |  | 0.001 |  | 0.002 | |
| The Dietary OBS was converted from a continuous variable to a categorical variable (quartiles). Data are presented as OR (95% CI). Crude model was adjusted with no covariates. Model 1 was adjusted for age, gender, and race. Model 2 included additional adjustments for education level, marital status, and PIR. Model 3 further adjusted for hypertension, high cholesterol, cancer, and diabetes. | | | | | | | | |  |

**Supplemental table 5.** Association of Lifestyle Oxidative Balance Score with Muscular Dystrophies.

| Muscular Dystrophies | Odds Ratio (95%CI); *p*-value | | | | | | | |
| --- | --- | --- | --- | --- | --- | --- | --- | --- |
|  | Crude model | | Model 1 | | Model 2 | | Model 3 | |
| Continuous | 0.73 (0.66, 0.79) | <0.001 | 0.69 (0.63, 0.76) | <0.001 | 0.71 (0.65, 0.77) | <0.001 | 0.72 (0.66, 0.79) | <0.001 |
| Q1 | 1.00 (ref) | | 1.00 (ref) | | 1.00 (ref) | | 1.00 (ref) | |
| Q2 | 0.74 (0.53, 1.04) | 0.079 | 0.70 (0.49, 1.01) | 0.057 | 0.74 (0.52, 1.06) | 0.100 | 0.77 (0.53, 1.11) | 0.152 |
| Q3 | 0.53 (0.35, 0.80) | 0.003 | 0.48 (0.31, 0.75) | 0.002 | 0.51 (0.33, 0.79) | 0.003 | 0.52 (0.33, 0.82) | 0.006 |
| Q4 | 0.27 (0.18, 0.40) | <0.001 | 0.23 (0.15, 0.35) | <0.001 | 0.25 (0.16, 0.37) | <0.001 | 0.26 (0.17, 0.40) | <0.001 |
| *p* for trend |  | <0.001 |  | <0.001 |  | <0.001 |  | <0.001 |
| The Lifestyle OBS was converted from a continuous variable to a categorical variable (quartiles). Data are presented as OR (95% CI). Crude model was adjusted with no covariates. Model 1 was adjusted for age, gender, and race. Model 2 included additional adjustments for education level, marital status, and PIR. Model 3 further adjusted for hypertension, high cholesterol, cancer, and diabetes. | | | | | | | | |

# Supplementary Figures


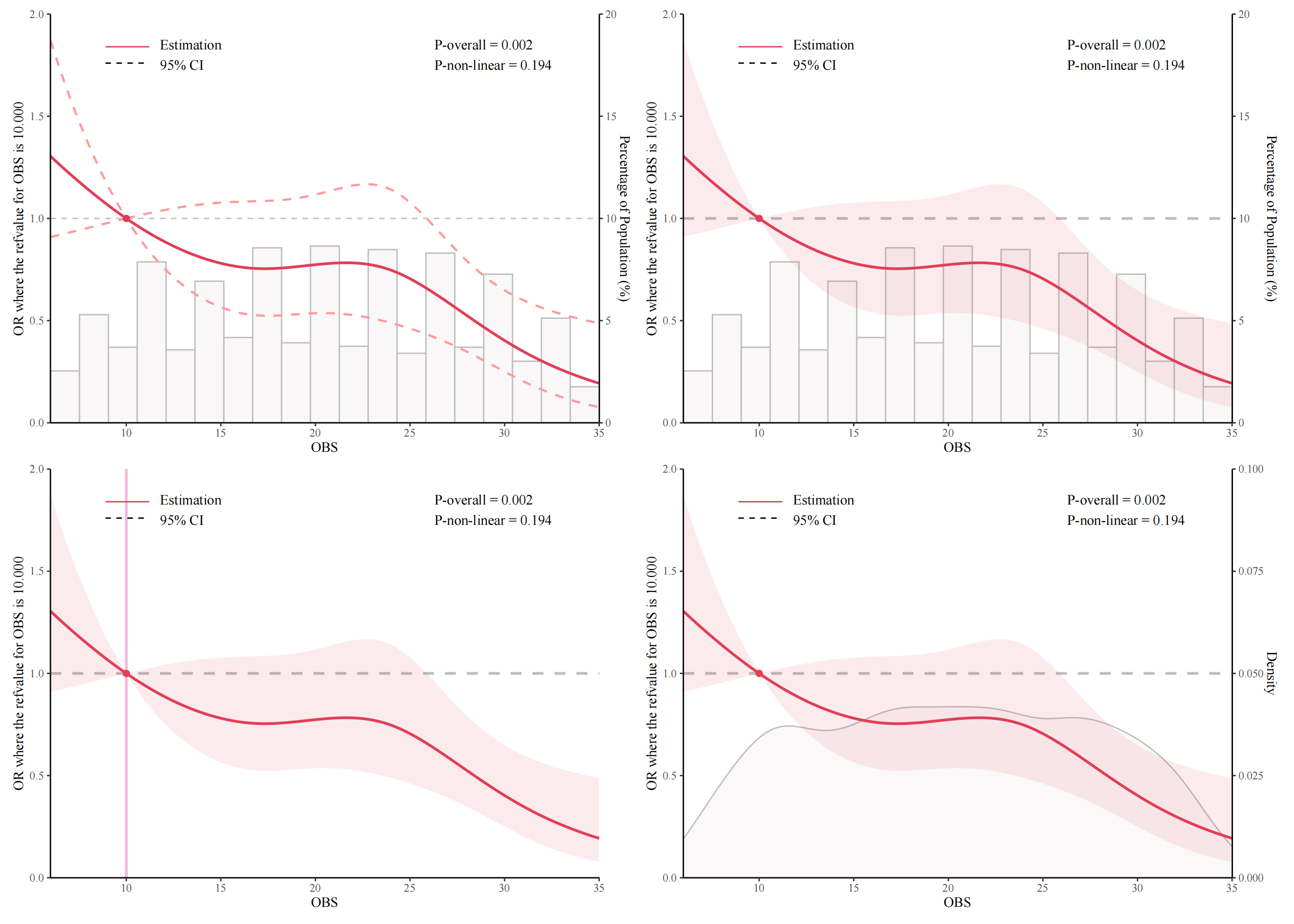


**Supplemental figure 1.** RCS analysis of the association between OBS and Muscular Dystrophies with number, bar chart of proportion, and density in male population. The association was adjusted for age, gender, race, education level, marital status, poverty income ratio, hypertension, high cholesterol, cancer, and diabetes. The median OBS was chosen as the reference. RCS, restricted cubic spline; OBS, oxidative balance score.


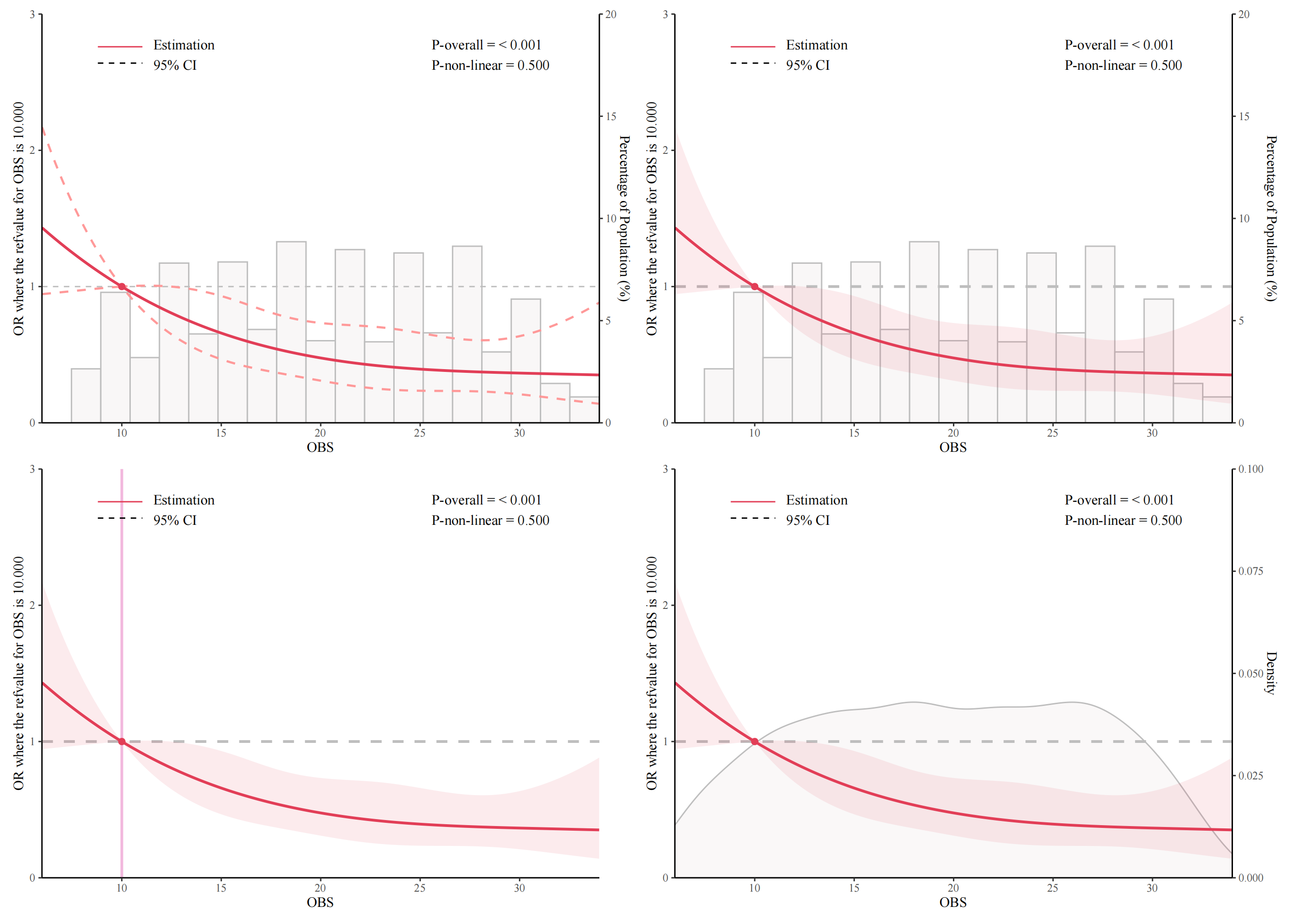


**Supplemental figure 2.** RCS analysis of the association between OBS and Muscular Dystrophies with number, bar chart of proportion, and density in female population. The association was adjusted for age, gender, race, education level, marital status, poverty income ratio, hypertension, high cholesterol, cancer, and diabetes. The median OBS was chosen as the reference. RCS, restricted cubic spline; OBS, oxidative balance score.
